# Enhancing Extracellular Adenosine Levels Restores Barrier Function in Acute Lung Injury Through Expression of Focal Adhesion Proteins

**DOI:** 10.3389/fmolb.2021.636678

**Published:** 2021-03-10

**Authors:** Wei Wang, Ning-yuan Chen, Dewei Ren, Jonathan Davies, Kemly Philip, Holger K. Eltzschig, Michael R. Blackburn, Bindu Akkanti, Harry Karmouty-Quintana, Tingting Weng

**Affiliations:** ^1^ Department of Thoracic Surgery, Renmin Hospital of Wuhan University, Wuhan, China; ^2^ Department of Biochemistry and Molecular Biology, McGovern Medical School, The University of Texas Health Science Center at Houston, Houston, TX, United States; ^3^ Houston Methodist J.C. Walter Jr. Transplant Center, Houston Methodist Hospital, Houston, TX, United States; ^4^ Division of Neonatal-Perinatal Medicine, Department of Pediatrics, Baylor College of Medicine, Houston, TX, United States; ^5^ Department of Anesthesiology, McGovern Medical School, The University of Texas Health Science Center at Houston, Houston, TX, United States; ^6^ UTHealth Pulmonary Center of Excellence, Houston, TX, United States; ^7^ Divisions of Critical Care, Pulmonary and Sleep Medicine, Department of Internal Medicine, McGovern Medical School, The University of Texas Health Science Center at Houston, Houston, TX, United States

**Keywords:** barrier function, pulmonary edema, ARDS, focal adhesion, equilibrative nucleoside transporters (ENTs), alveolar type 2 cell

## Abstract

**Background:** Acute respiratory distress syndrome (ARDS) is a clinical presentation of acute lung injury (ALI) with often fatal lung complication. Adenosine, a nucleoside generated following cellular stress provides protective effects in acute injury. The levels of extracellular adenosine can be depleted by equilibrative nucleoside transporters (ENTs). ENT inhibition by pharmaceutical agent dipyridamole promotes extracellular adenosine accumulation and is protective in ARDS. However, the therapeutic potential of dipyridamole in acute lung injury has not yet been evaluated.

**Methods:** Adenosine acts on three adenosine receptors, the adenosine A1 (Adora1), A2a (Adora2a), the A2b (Adora2b) or the adenosine A3 (Adora 3) receptor. Accumulation of adenosine is usually required to stimulate the low-affinity Adora2b receptor. In order to investigate the effect of adenosine accumulation and the contribution of epithelial-specific ENT2 or adora2b expression in experimental ALI, dipyridamole, and epithelial specific ENT2 or Adora2b deficient mice were utilized. MLE12 cells were used to probe downstream Adora2b signaling. Adenosine receptors, transporters, and targets were determined in ARDS lungs.

**Results:** ENT2 is mainly expressed in alveolar epithelial cells and is negatively regulated by hypoxia following tissue injury. Enhancing adenosine levels with ENT1/ENT2 inhibitor dipyridamole at a time when bleomycin-induced ALI was present, reduced further injury. Mice pretreated with the ADORA2B agonist BAY 60-6583 were protected from bleomycin-induced ALI by reducing vascular leakage (558.6 ± 50.4 vs. 379.9 ± 70.4, *p* < 0.05), total bronchoalveolar lavage fluid cell numbers (17.9 ± 1.8 to 13.4 ± 1.4 e4, *p* < 0.05), and neutrophil infiltration (6.42 ± 0.25 vs. 3.94 ± 0.29, *p* < 0.05). While mice lacking *Adora2b* in AECs were no longer protected by dipyridamole. We also identified occludin and focal adhesion kinase as downstream targets of ADORA2B, thus providing a novel mechanism for adenosine-mediated barrier protection. Similarly, we also observed similar enhanced ADORA2B (3.33 ± 0.67 to 16.12 ± 5.89, *p* < 0.05) and decreased occludin (81.2 ± 0.3 to 13.3 ± 0.4, *p* < 0.05) levels in human Acute respiratory distress syndrome lungs.

**Conclusion:** We have highlighted a role of dipyridamole and adenosine signaling in preventing or treating ALI and identified Ent2 and Adora2b as key mediators in important for the resolution of ALI.

## Introduction

Acute respiratory distress syndrome (ARDS) is a clinical presentation of acute lung injury (ALI) characterized by progressive arterial hypoxemia, and dyspnea (Nuckton et al., 2002) caused primarily by pulmonary edema (Ware and Matthay, 2000; Matthay et al., 2012). According to the Berlin definition, ARDS is the presence of bilateral opacities from chest imaging, pulmonary edema not due to cardiac failure or fluid overload, and arterial oxygenation calculated by the PaO_2_/FiO_2_ ratio. A ratio less than 300 mmHg is termed mild, less than 200 is termed moderate, and less than 100 is severe (Ranieri et al., 2012). The etiology of ARDS is complex and multifactorial: sepsis, pneumonia, major trauma, blood transfusion, smoke inhalation, and aspiration of salt water, fresh water or gastric contents can all promote ALI (Ware and Matthay, 2000). ARDS affects approximately 200,000 Americans and has a mortality of approximately 45% (Flori et al., 2005; ). Currently there are no effective treatment options for ARDS (Matthay and Zemans, 2011). This has been painfully apparent in the current COVID-19 pandemic where the vast majority of patients that succumb present with an atypical form of ARDS (Gong et al., 2020; Li and Ma, 2020; Liang et al., 2020), this underscores our need to develop new treatment options for this devastating disease.

Disruption of epithelial and endothelial barrier function is a hallmark of ALI (Ware, 2006). The lung epithelium usually maintains an impermeable network through interactions of numerous structural proteins, including tight junction and focal adhesion proteins. Disruption of tight junction and focal adhesion proteins, including occludins, claudins and focal adhesion kinase (FAK), in epithelial cells promotes the permeability of alveolar epithelium and facilitates the progression of ARDS (Ma et al., 2013; Suzuki, 2013). Preserved epithelial barrier function is inversely associated with mortality of patients with ARDS (Matthay and Zemans, 2011; Fanelli and Ranieri, 2015), suggesting signaling pathways regulating the expression and/or functions of the tight junction and focal adhesion proteins may be a valid therapeutic target for ARDS.

Recently, several studies suggest extracellular adenosine as a potential therapeutic agent for the prevention of lung damage (Kong et al., 2006; Eckle et al., 2009; Eltzschig, 2009; Eltzschig et al., 2013; Karmouty-Quintana et al., 2013). Extracellular adenosine is enhanced in areas with injury and is generated by the breakdown of precursor nucleotides including ATP, ADP and AMP by nucleotidases CD39 and CD73 (Eltzschig et al., 2013; Karmouty-Quintana et al., 2013; Idzko et al., 2014). Adenosine functions through activation of G-protein-coupled receptors (Adora1, Adora2a, Adora2b, and Adora3), and increased extracellular adenosine is normally associated with enhanced barrier functions and decreased ALI (Blackburn et al., 2009; Eckle et al., 2009).Blocking equilibrative nucleoside transporters (ENTs) with dipyridamole or by genetically deleting *Ent2* significantly enhanced extracellular adenosine in the alveoli, and as a result suppressed pulmonary inflammation and vascular leakage in ventilator-induced lung injury (Eckle et al., 2013; Morote-Garcia et al., 2013). In contrast, genetic depletion of CD73, a nucleotidase that is essential for adenosine generation, increased inflammation as well as pulmonary vascular leakage (Eltzschig et al., 2005; Reutershan et al., 2009; Hasko et al., 2011; Thompson et al., 2011). However, the therapeutic potential of enhancing extracellular adenosine levels at a time when acute injury is already established has not yet been evaluated. In addition, the mechanisms of how extracellular adenosine regulates the integrity of pulmonary epithelium and endothelium are not well understood.

Several mouse models of ALI has been developed, including mechanical ventilation, sepsis, acid aspiration, and ischemia-reperfusion induced ALI (Rosenthal et al., 1998; Matute-Bello et al., 2008). However, none of them truly recapitulate features of human ARDS. Intratracheal (i.t.) bleomycin is one of the most commonly used models of ALI in mice. Bleomycin is a chemotherapeutic agent derived from *Streptomyces* verticillus, and it is clinically used to treat a variety of human cancers. However, one of the adverse effect of bleomycin therapy is lung injury and pulmonary fibrosis (Froudarakis et al., 2013; Martin W. G. et al., 2005). Intratracheal bleomycin induced ALI reproduces many features of ARDS, including neutrophil infiltration and increased levels of pro-inflammatory cytokines such as interleukin (IL)-1β, IL-6, IL-1R1, keratinocyte chemoattractant (KC), tumor necrosis factor (TNF)-A at the early inflammatory stage (Matute-Bello et al., 2008; Williamson et al., 2015). Furthermore, profibrotic cytokines such as Granulocyte colony-stimulating factor (G-CSF), Granulocyte-macrophage colony-stimulating factor (GM-CSF), interferon (IFN)-γ, and IL-4 and the subsequent development of fibrosis at the late stage (Matute-Bello et al., 2008; Williamson et al., 2015). This acute and chronic phases of i.t.- bleomycin instillation reflect the acute and chronic phases of ARDS in human that is characterized by and acute inflammatory onset, hypoxemia and subsequent fibrotic tissue deposition (Matthay and Zemans, 2011).

Accordingly, in the present study, using the intratracheal bleomycin-induced ALI model, we demonstrate that elevating adenosine levels acutely is protective against ALI, both prophylactically and therapeutically. We also demonstrate that activation of Adora2b is important to restore barrier function by modulating the expression of focal adhesion proteins.

## Materials and Methods

### Generation and Genotyping of Mouse Lines

All of the animal experiments were reviewed and approved by the Animal Welfare Committee at the University of Texas Health Science Center at Houston. *Ent2* ^*f/f*^ C57BL6 mice were gifts from Dr Eltzschig’s lab at the University of Colorado Denver (Eckle et al., 2013; Grenz et al., 2012a). Conditional SPC-CreER-*Ent2* ^*f/f*^ and SPC-CreER-*Adora2b* ^*f/f*^ mice were obtained by crossing *Ent2* ^*f/f*^ and *Adora2b* ^*f/f*^ with SPC-CreER mice. Age and gender matched littermate SPC-CreER mice used as controls for SPC-CreER-*Ent2* ^*f/f*^ and SPC-Cre-*Adora2b* ^*f/f*^ *.* All of the mice were housed in a pathogen-free environment with adequate food and water. No evidence of fungal, parasitic, or bacterial infection was observed.

### Mouse Treatment

The SPC-CreER-*Ent2* ^*f/f*^, SPC-CreER-*Adora2b* ^*f/f*^ and control SPC-CreER were injected intraperitoneally (i.p.) with ∼75 mg/kg tamoxifen once every 24 h for a total of five consecutive days to induced the cre recombination. A week after the last tamoxifen injection, mice were injected intratracheally with bleomyicn as previously described (Zhou et al., 2009). Briefly, 10–12 week old female C57BL/6 mice were anesthetized with freshly made avertin (250 mg/kg) and instilled intratracheally with saline or 2.5 U/kg bleomycin (Teva Parenteral Medicine, Irvine, CA). The lungs and bronchoalveolar lavage fluid (BAL) were collected for analysis on day 3 or day 7 after bleomycin injection.

Dipyridamole (Sigma-Aldrich, St. Louis, MO) was diluted in 400 ul DMSO, then 2 ml 100% ethanol and then 18 ml sterile corn oil to a final concentration of 1 mg/ml. For the therapeutic model, two days after bleomycin injection, mice were injected i.p. with 5 mg/kg dipyridamole (Sigma-Aldrich, St. Louis, MO) twice daily or with 500 μg/kg BAY 60-6583 (Tocris, Minneapolis, MN) daily (Wang et al., 2013; Zimmerman et al., 2013). Control mice were injected with same volume of vehicle. The lungs and bronchoalveolar lavage fluid (BAL) were collected for analysis on day 7. To avoid animal death, only female mice were used in this study because male mice are more vulnerable to i.t. bleomycin-induced fibrosis.

### Evans Blue Measurement of Vascular Permeability

Mice were i.p. injected with 0.2 ml Evans blue dye (0.5% in PBS) (17). Four hours after dye administration, mice were anesthetized with freshly made avertin (250 mg/kg) and lungs were exposed and perfused with PBS. Lungs were then isolated, weighted and incubated in 1 ml formamide overnight at 55°C to extract dye. Dye concentrations were measured spectrophotometrically at 610 nm. The content of Evans blue dye was determined by generating a standard curve from dye dilutions.

### Quantitative Real-Time PCR

Cells or lung tissues were lyzed using TRIZOL Reagent (Thermo Fisher Scientific, Fair Lawn, NJ, United States), and total RNA were extracted using RNeasy mini Kit (Qiagen). Then RNA were treated with Heat&Run gDNA remover kit (ArcticZymes) and reverse transcripted into cDNA using Transcriptor First strand cDNA synthesis (Roche). Real-time PCR was carried out using LightCycler 96 using the following primers: Mouse *Ent1* forward: 5′- CTT​GGG​ATT​CAG​GGT​CAG​AA-3′, Mouse *Ent1* reverse: 5′- ATC​AGG​TCA​CAC​GAC​ACC​AA -3′; Mouse *Ent2* forward: 5′- CAT​GGA​AAC​TGA​GGG​GAA​GA -3′, Mouse *Ent2* reverse: 5′- GTT​CCA​AAG​GCC​TCA​CAG​AG-3′; Mouse β-Actin forward: 5′- GGC​TGT​ATT​CCC​CTC​CAT​CG-3′; Mouse β-Actin forward: 5′- CCA​GTT​GGT​AAC​AAT​GCC​ATG​T -3′. Data was quantified using the comparative Ct method and presented as mean ratio to β-actin.

### BAL Collection and Differential Cell Count

Mice were anesthetized with avertin, the trachea was exposed and lavaged four times with 0.3 ml PBS containing 10 µM dipyridamole, 10 µM αβ-methylene ADP, and 10 µM 5′-deoxycoformycin (Sigma-Aldrich), which will pool about 1 ml bronchoalveolar lavage (BAL) fluid. Total number of BAL cells was counted using a hemocytometer. Cellular differentials were determined by cytospinning BAL aliquots onto microscope slides and staining with Diff-Quick (Dade Behring, Deerfield, IL).

### Adenosine Measurement

Adenosine concentration in BAL fluid was measured as described previously (45). Briefly, BAL fluid was centrifuged at highest speed to clear cells and debris. 200 μl BAL supernatant was analyzed by reverse-phase high-performance liquid chromatography (HPLC) as described previously (45). Representative peaks were identified and quantified using external standard curves.

### Albumin Measurement

The albumin level in BAL was determined using the mouse albumin ELISA kit (Immunology Consultants Laboratory, Inc., Portland, OR, United States). Briefly, BAL samples and standards were added to predesignated wells coated with anti-albumin antibody for 30 min. After the unbound proteins were rinsed, appropriately diluted Enzyme antibody was added and incubated for an additional 30 min. Then the wells were rinsed again and incubated with TMB Substrate Solution for 10 min. Finally, the reactions were stopped by adding Stop Solution to each well. The absorbance (450 nm) of the contents of each well was measured by plate reader and albumin concentration was calculated based on the standard curve.

### Cell Culture and Hypoxia Exposure

MLE12 cells (ATCC) were cultured in RPMI Media 1,640 (Life Technologies, Grand Island, NY, United States) containing 10% fetal bovine serum and 100 U/ml penicillin G, and 100 μg/ml. RLE-6 TN was purchased from ATCC and grown in Dulbecco’s Modified Eagle’s medium, nutrient mixture F-12 Ham (1:1) supplemented with 10% fetal bovine serum, 40 mmol/L HEPES, 100 U/ml penicillin G, and 100 μg/ml. To study the role of Adora2b on junction proteins and transepithelial electric resistance, cells were serum-starved overnight, treated with 10 μg/ml ADA (Roche) for 30 min, followed by incubation in 500 nM BAY 60-6583, and/or 1 µM MRS 1754, and/or 10 μM H89, then the cells were placed in a sealed hypoxia chamber and exposed to 2% oxygen supplied with 95% N_2_ and 5% CO_2_ for 1–6 h.

To measure calpain activity, cells were serum-starved, treated with different compounds and exposed to normal air or 2% oxygen for 1 h. Calpain activities were determined using the Calpain-Glo™ protease Assay Kit (Promega, Madison, WI, United States). Experiments were repeated 3 times and triplicated.

### Transepithelial Electric Resistance (TEER) Measurement

RLE-6TN cells were seeded on Transwell inserts (0.4-μm pore size, 12-mm inside diameter; Corning Incorporated, Corning, NY) at a density of 1 × 10^5^ cells/cm^2^. TEER was measured by EVOM2 (World Precision Instruments, Sarasota, FL, United States). When the electrical resistance between the apical and basolateral surfaces of the monolayers was >400 Ω/cm^2^, cells were serum-starved overnight, treated with 10 μg/ml ADA for 30 min, followed by incubation in 1 nM BAY 60-6583, and/or 100 nM MRS 1754, and/or 10 μM H89, then the cells were placed in a sealed hypoxia chamber and exposed to 2% oxygen supplied with 95% N_2_ and 5% CO_2_. TEER was measured 0, 0.5, 1, 2, 4 and 6 h after hypoxia exposure.

### Western Blot

Cells or lung samples were collected and lysed in RIPA lysis buffer (50 mM Tris-HCl PH 7.4, 150 mM NaCl, 1% NP-40) containing both protease and phosphatase inhibitor cocktail (Thermo Fisher Scientific, Fair Lawn, NJ, United States). Western blots were carried out as previously described using primary rabbit anti-ENT1 (Abgent, San Diego, CA, United States), rabbit anti-ENT2 (Abcam), rabbit anti-occludin, rabbit anti-phosphorylated FAK, rabbit anti FAK, rabbit anti ZO-1, rabbit anti claudin-1 (Cell signaling, Boston, MA, United States), mouse anti-CD73 antibodies (ThermoFisher Scientific), mouse anti-Hif-1α antibodies (Novus Biological, Littleton, CO, United States), or mouse anti-β-Actin antibodies (Sigma) and corresponding secondary antibodies conjugated to horseradish peroxidase (Jackson ImmunoResearch, West Grove, PA, United States). Finally, the membranes were developed with Pierce ECL Western Blotting Substrate (Thermo Fisher scientific, Fair Lawn, NJ, United States).

### H&E Staining and Immunohistochemistry

Mouse lungs were fixed in 10% formaldehyde for at least 24 h. Lungs were then dehydrated, paraffin embedded and sectioned at a thickness of 5 µm. For Hematoxylin and Eosin (H&E), staining sections were rehydrated, incubated with Hematoxylin Solution, Gill No. 3 (Sigma) and then incubated with aqueous 1% Eosin Y solution (Sigma). Finally, slides were dehydrated and mounted in Cytoseal™ XYL (ThemoFisher scientific, Waltham, MA, United States).

For ENT1, ENT2 and neutrophil staining, sections were rehydrated, quenched with 3% hydrogen peroxide, incubated in citric buffer (VectorLabs, Burlingame, CA, United States) for antigen retrieval, and blocked with Avidin/Biotin Blocking System (VectorLabs) and then 5% normal goat serum. After that, the sections were incubated with anti-ENT1, ENT2 (1:200, Abcam), and Anti-Neutrophil antibody NIMP-R14 (1:500, rabbit polyclonal, Abcam, Cambridge, MA, United States) overnight at 4°C. Slides were then incubated with appropriate secondary antibodies (1:1,000, VectorLabs) for 1 h and ABC Elite streptavidin reagents for 30 min at room temperature. Finally, slides were developed with Vector blue (VectorLabs) or 3,3-diaminobenzidine (Sigma-Aldrich, St. Louis, MO, United States) and counter-stained with methyl green. Stained slides were blindly counted for the numbers of positive stained neutrophils at ×20 objective. Ten random areas per lung were analyzed to get the final average number of infiltrated neutrophil.

### Human Lung Collection

Normal and ARDS lungs were purchased from the International Institute for the Advancement of Medicine (IIAM) Usage of these tissues was performed under IRB: HSC-MS-08-0354. Discarded lungs for transplantation were considered normal if they did not have a history of chronic lung injury, presented with a partial pressure of oxygen (PaO_2_) fraction of inspired oxygen (FIO_2_) ratio PaO_2_/FIO_2_ over 300 mmHg and the absence of bilateral opacities by chest imaging. Discarded lungs for transplantation that met the Berlin definition of ARDS: presence of bilateral opacities from chest imaging, pulmonary edema not due to cardiac failure or fluid overload and arterial oxygenation calculated by the PaO_2_/FiO_2_ ratio less than 300 mmHg (Ranieri et al., 2012), were used for our study. Samples from four normal and three ARDS lungs were analyzed.

### CD73 Activity Assay

Pulverized normal and ARDS lungs were lysed in NP-40 lysis buffer (1% NP-40, 150 mM NaCl, 50 mM Tris-Cl, PH 8.0) containing protease inhibitor cocktail (ThermoFisher). Protein concentration were determined using the Pierce™ BCA protein assay kit (ThermoFisher). For each assay, five ug lung lysate was mixed with assay buffer (10 mM HEPES, 10 mM MgCl_2_, 10 µM DCF and 200 mM AMP), and immediately incubated at 37°C for 30 min. At the end of the incubation, reaction was inactivated by incubating the mixture at 95°C for 5 min. Adenosine levels were measured using HPLC as described above. The activites of CD73 were integrated as the amount of adenosine synthesized per minutes per Gram of total protein.

### Statistical Analysis

For scientific rigor, all *in vitro* cell culture experiments were repeated at least three times and each treatment was at least duplicated. For experiments having no more than two treatment groups, two-tailed student t-tests were used to assess whether the means of two groups were statistically different from each other. Otherwise, one-way or two-way ANOVA were used to determine whether there were significant differences among treatments. If so, Tukey’s multiple comparison test were carried out to determine whether there were significant differences between any of the two treatments. The data were considered significant with *p* < 0.05. All results were presented as Mean ± SEM to indicate variability.

## Results

### The Expression of ENT2, but not ENT1, is Negatively Regulated by Hypoxia Following Tissue Injury

**FIGURE 1:**
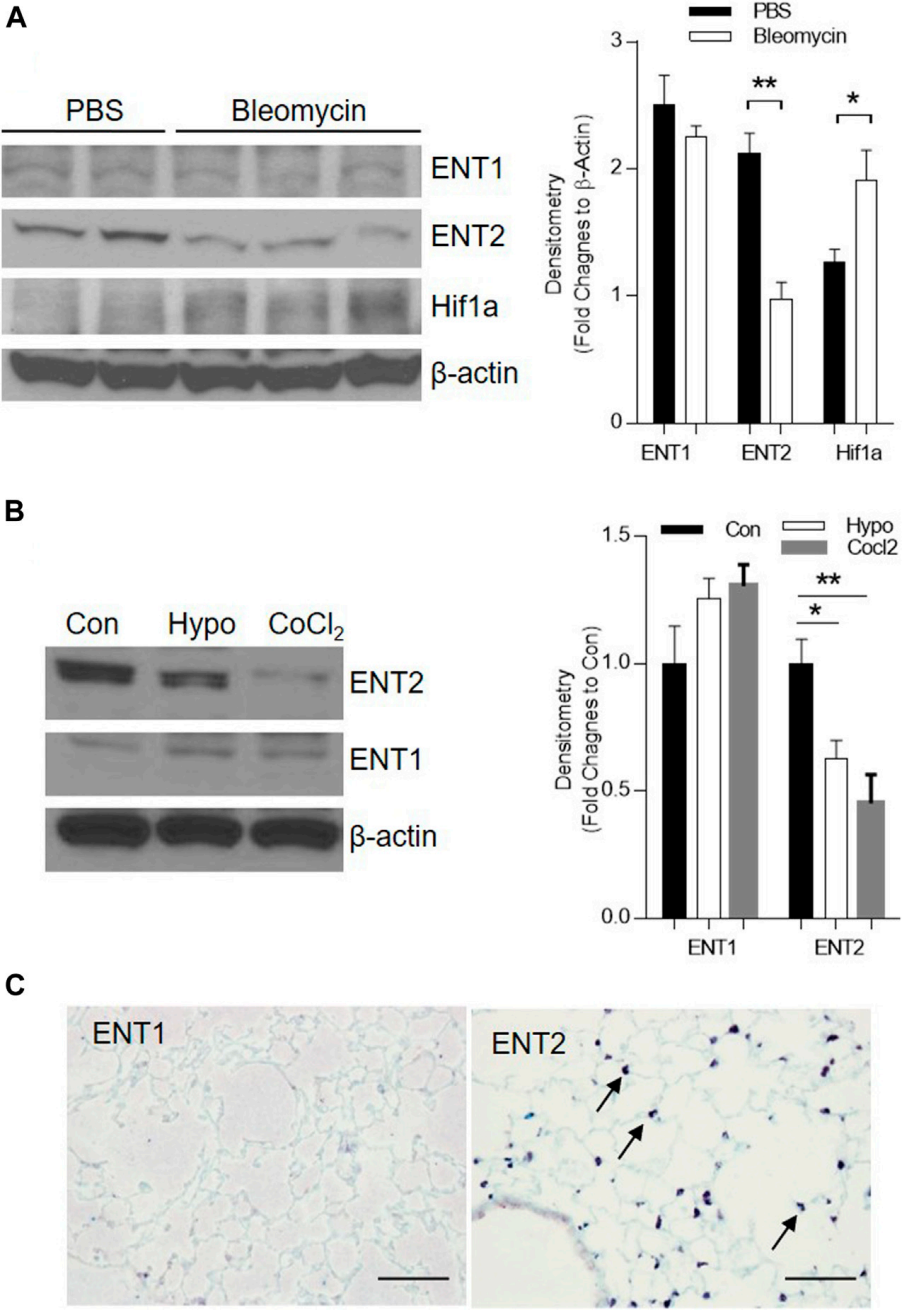
ENT2 expression is decreased in bleomycin-induced ALI. C57BL6 mice were i.t. injected with 2.5 U/kg bleomycin and lungs were collected for analysis on day 3. **(A)** Left Panel: The protein expression of ENT1, ENT2, and Hif1a were determined using western blot. β-Actin was used as an internal control. Right Panel: Densitometry of the Western Blot image were analyzed and data were presented as fold changes to β-Actin ± MSE. *p < 0.05, **p < 0.01. **(B)** Primary alveolar epithelial type II (AECII) were isolated from normal mouse lungs and exposed to 2% oxygen, or 100 mM CoCl_2_ for 24 h. Right Panel: Densitometry of the Western Blot image were analyzed and data were presented as fold changes to Control (Con) ± MSE. *p < 0.05, **p < 0.01. **(C)** Cellular localization of ENT1 and ENT2 (brown, arrow) was visualized using immunohistochemistry in a normal healthy mouse lung. The protein levels of ENT1, ENT2, and β-Actin were determined using western blot. n = 3, *p < 0.05 vs. control. Scale bar = 100 µm.

Extracellular adenosine is generated following injury and can be transported by ENTs into the cells and then depleted in the extracellular space (Karmouty-Quintana et al., 2013). To understand whether ENT expression is suppressed following bleomycin-induced acute lung injury and partially contributes to the increase of extracellular adenosine levels, we examined the transcript and protein expression of ENTs in the lungs of mice instilled with bleomycin (i.t.) after 3 days. ENT2 protein levels were decreased in the lungs of mice treated with bleomycin (Figure 1A), suggesting that ENT2 might be the transporter which is more actively regulated following lung injury. Previous studies have demonstrated that hypoxia is one of the important regulators of ENTs (Eckle et al., 2013). Indeed, we observed an increased expression of hypoxia-inducible factor 1 alpha (Hif-1a) following bleomycin treatment (Figure 1A), suggesting that hypoxia is present in the injured lungs and might be the mechanism that leads to decreased ENT expression. To further understand whether hypoxia directly regulates ENT expression, we isolated primary alveolar epithelial type II cells (AECII) from mice and exposed them to 2% oxygen or CoCl_2_, a chemical inducer of Hif-1a. Consistent with the results we observed using whole lung lysates, ENT2 but not ENT1 expression was dramatically down-regulated by both hypoxia and CoCl_2_ (Figure 1B), suggesting that hypoxia following acute lung injury can suppress ENT2 expression in a Hif-1a dependent manner. Immunohistochemistry demonstrated that ENT2 is mainly located in alveolar epithelial cells (arrow) in normal mouse lungs (Figure 1C). Taken together, our findings demonstrate that ENT2, but not Ent1, is repressed by hypoxia during ALI induced by bleomycin.

### ENT2 Is the Major Transporter to Regulate BAL Adenosine Levels

**FIGURE 2:**
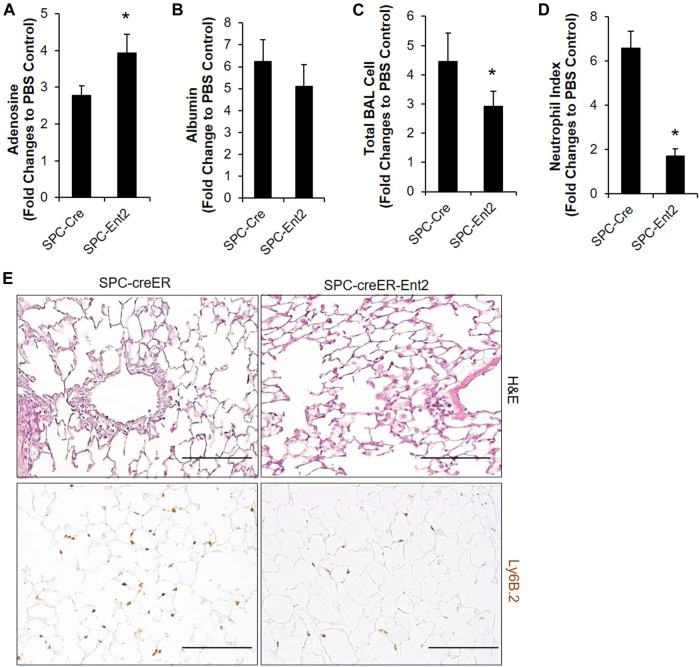
Bleomycin-induced ALI is attenuated in mice with epithelial specific deletion of Ent2. SPC-CreER- Ent2−/− mice or littermate SPC-CreER controls were i.p. injected with 75 mg/ml tamoxifen daily for 5 days. One week after the first tamoxifen injection, mice were **I**.t. injected with 2.5 U/kg bleomycin. On day 3, BAL adenosine **(A)** and albumin levels **(B)**, and total BAL cell number **(C)** were analyzed. **(D)** Neutrophil infiltration was determined by staining with anti-Ly6B.2, and cell number per high powered filed were counted. **(E)** Representative H&E and Ly6b staining showing pulmonary inflammation and neutrophil infiltration in different groups of lungs. n ≥ 4, *p < 0.05, **p < 0.01 vs. Control. #*p* < 0.05 vs. wild type bleomycin. Scale bar = 100 µm.

Our immunostaining mainly located ENT2 in alveolar epithelial cells, suggesting that alveolar epithelial cells might be the key cells to mediate the ENT2 regulation of extracellular adenosine levels. To define this, we crossed SPC-CreER mice with mice carrying a floxed allele of *Ent2* to generate transgenic mice with *Ent2* deficient specifically in alveolar epithelial cells. Seven days after inducing the cre recombinase with tamoxifen, SPC-CreER-*Ent2* mice were administrated with bleomycin and compared with SPC-CreER controls. SPC-CreER-*Ent2* mice had significantly enhanced extracellular adenosine levels (Figure 2A) in association with attenuated vascular leakage (Figure 2B) and inflammatory cell infiltration (Figure 2C) compared to controls. Further differential cell counts revealed that SPC-CreER-*Ent2* mice presented with reduced neutrophil cell infiltration (Figure 2D,E). In summary, these findings demonstrate a selective protective role for epithelial ENT2 in bleomycin-induced lung injury.

### Adenosine Through Adora2b Is Important to Protect Lungs From Acute Injury

We have demonstrated that elevation of extracellular adenosine is limited by epithelial ENT2 and is associated with increased protection from lung injury. Next, we want to investigate which adenosine receptor is involved in this protection. Previous studies in a mouse model of VILI indicate that adenosine, through the Adora2b receptor, is the major pathway that promotes barrier protection (Eckle et al., 2013). Based on these notions, we hypothesize that Adora2b is the major adenosine receptor that mediates this protection.

**FIGURE 3:**
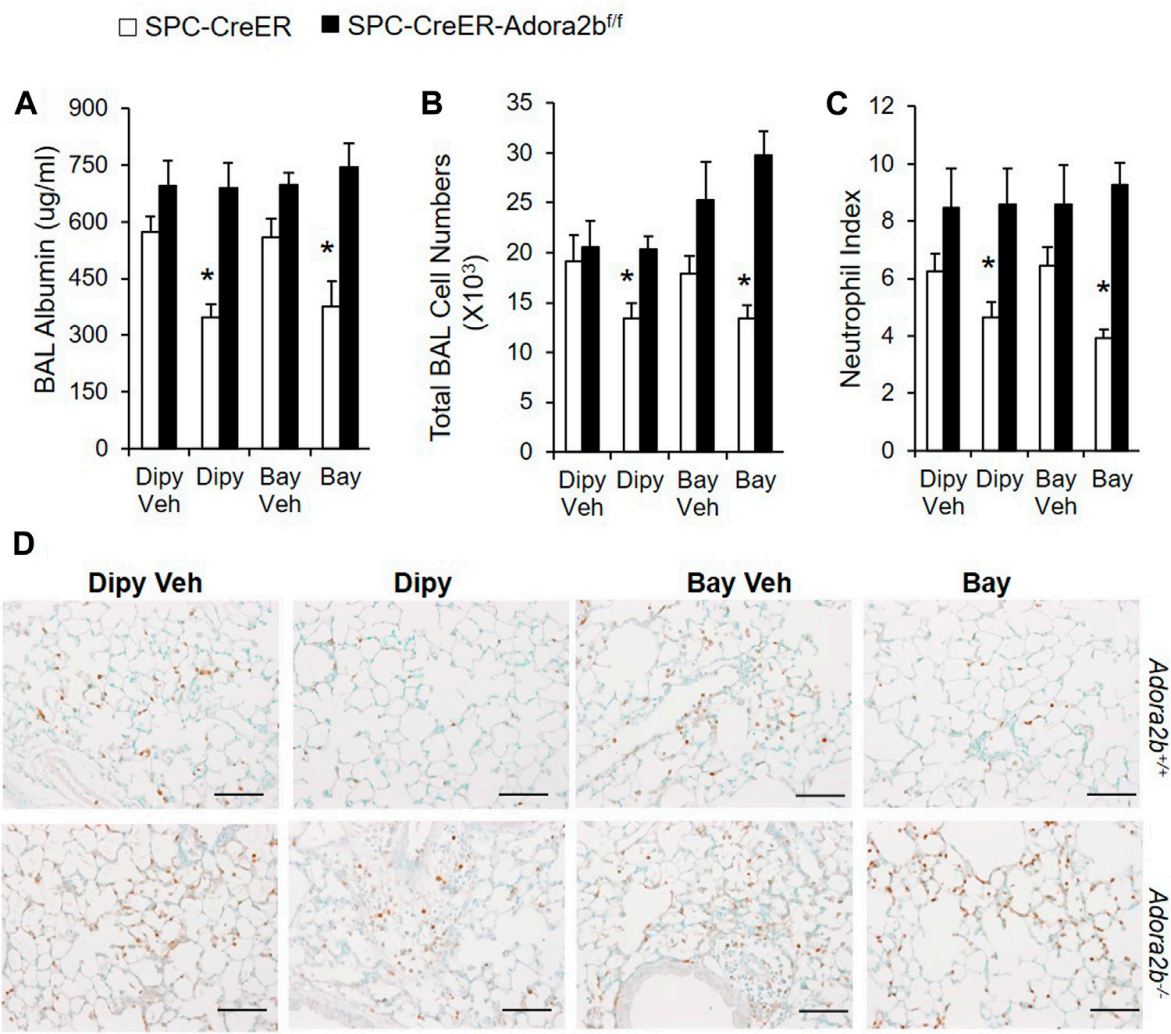
Bleomycin-induced ALI in mice with Adora2b deletion specially in alveolar epithelial cells. SPC-Cre-Adora2b−/− mice and littermate controls were I.p. injected with 75 mg/ml tamoxifen daily for 5 days to induce Cre recombination. One week after the first tamoxifen injection, mice were pretreated with vehicle control, 5 mg/kg dipyridamole or 500 ug/kg BAY 60-6583 1 h before I.t. injected with 2.5 U/kg bleomycin and then continuously injected with dipyridamole twice daily or BAY 60-6583 once daily until day 3 for analysis. **(A)** BAL albumin levels were measured to show vascular leakage. **(B)** Total BAL cell numbers were counted to determine pulmonary inflammation. **(C)** Neutrophil index was calculated by counting the number of neutrophil per high power field from slides stained with anti-Ly6B.2 antibody. n = 5, *p < 0.05 vs. corresponding Veh control using student t-test. **(D)** Representative lung sections stained with Ly6B.2 to show neutrophil infiltration. n ≥ 4, *p < 0.05 vs. vehicle using one way ANOVA followed by Bonferroni's multiple comparisons test. Scale bar = 100 µm.

Previous studies shown that Adora2b is mainly expressed in alveolar epithelial cells, and Adora2b in epithelial cells is essential to mediate the protective role of adenosine in ventilation induced acute lung injury (Cagnina et al., 2009; Eckle et al., 2013). To determine whether epithelial Adora2b is also important for the protection of barrier function in our model of acute lung injury, we generated mice with Adora2b deleted specially in alveolar epithelial type II cells by crossing SPC-creER mice with mice carrying a floxed allele of Adora2b, and induced ALI in these mice with bleomycin. Compared to SPC-Cre mice, in SPC-cre-*Adora2b* ^*f*/f^ mice, dipyridamole or BAY 60-6583 (an Adora2B agonist) treatment can no longer alleviate vascular leakage (Figure 3A), inflammation (Figure 3B,D), neutrophil infiltration (Figure 3C,D) after bleomycin exposure. Taken together, our study suggests that epithelial Adora2b is important to mediate the protective role of extracellular adenosine in bleomycin-induced acute lung injury.

### Inhibiting ENTs at a Time Point When Acute Lung Injury is Already Established Protects Lungs From Bleomycin-Induced Injury

**FIGURE 4:**
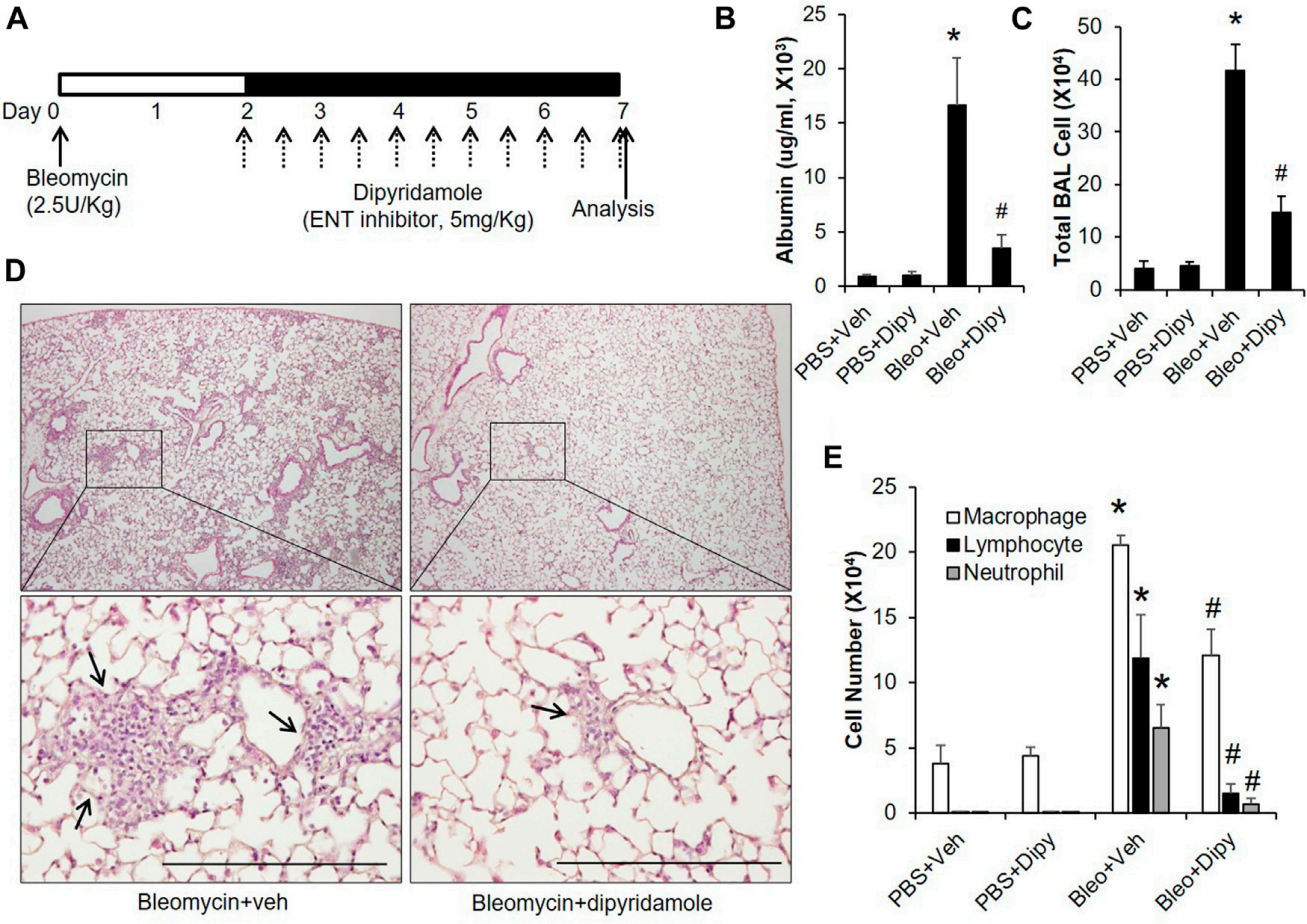
Therapeutic effect of dipyridamole on established ALI. **(A)** A diagram showing how mice were treated with dipyridamole and bleomycin. Mice injected with same volume of Vehicle were used as control. **(B)** BAL albumin levels were measured from different treatment groups. **(C)** BAL total cell numbers were counted. **(D)** Lungs from bleomycin and/or dipyridamole treated mice were sectioned and stained with H&E. Representative pictures were shown. Scale bar = 500 µM. **(E)** Differential assay was performed to determine the degree of macrophage, lymphocyte and neutrophil trafficking into the lungs. n ≥ 5, *p < 0.05 vs. PBS. #*p* < 0.05 vs. bleomycin using one way ANOVA followed by Bonferroni's multiple comparisons test. Scale bar = 200 µm.

Based on the above findings that extracellular adenosine plays a role in preventing bleomycin-induced injury, we next established another model to see whether adenosine is also beneficial in treating already established ALI. In this model, mice were intratracheally instilled with bleomycin; after 2 days, mice were i.p injected with dipyridamole twice daily until day 7 for analysis (Figure 4A). Dipyridamole significantly reduced vascular leakage (Figure 4B) as indicated by the amount of albumin leaking into the BAL. Pulmonary inflammation was also dramatically suppressed as shown by both total BAL cell count (Figure 4C) and H&E staining (Figure 4D). Next, we performed differential assays to understand which inflammatory cells were trafficking into the lungs. We observed that both lymphocytes and neutrophils, which are significantly induced by bleomycin, were repressed by dipyridamole treatment (Figure 4E). These findings indicate that inhibiting ENTs at a time point when acute lung injury is already established can also protect lungs from bleomycin-induced injury.

### Adenosine Through Adora2b is Important for Lungs to Recover From Acute Injury

**FIGURE 5:**
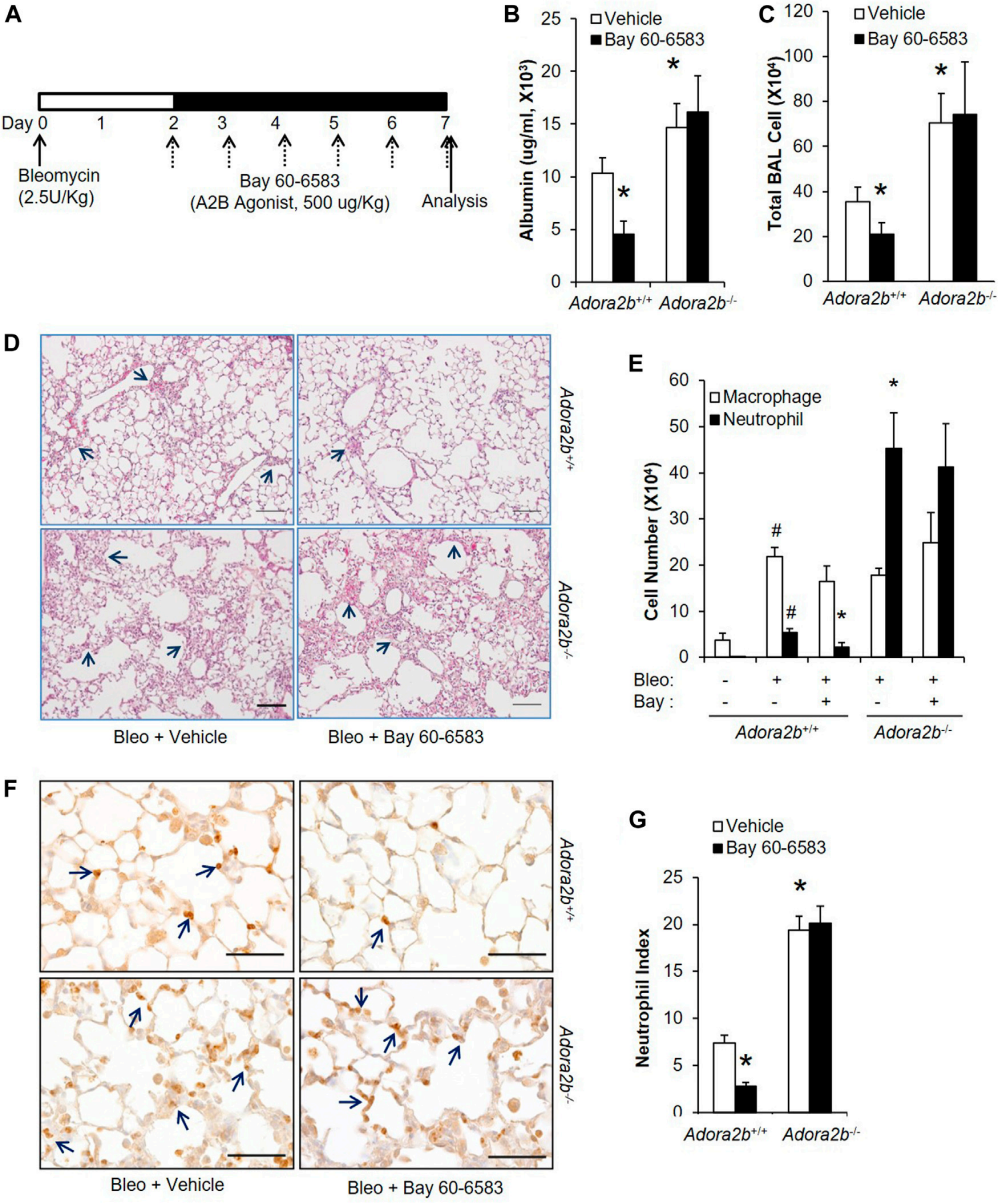
Therapeutic effect of BAY 60-6583 on established ALI in *Adora2b+/+* and *Adora2b−/−* mice. **(A)** A diagram showing how *Adora2b+/+* and *Adora2b−/−* mice were treated with BAY 60-6583 and bleomycin. **(B)** BAL albumin levels were measured to show the levels of vascular leakage. **(C)** BAL total cell number was counted. **(D)** Representative H&E stainings of lungs from bleomycin and/or dipyridamole treated mice. Arrows pointing areas with inflammation. Scale bar = 500 µM. **(E)** Differential assay was performed to determine the number of macrophage and neutrophil in the lungs. **(F)** Mouse lung sections from different treatment group were stained with anti-Ly6B.2 to visualize neutrophil expression and localization. Arrow: positive stained neutrophils. Scale bar = 100 µM. **(G)** The number of neutrophil for each treatment group was quantitated. N = 5, *p < 0.05 vs. wildtype bleomycin vehicle. #*p* < 0.05 vs. wild type PBS using one way ANOVA followed by Bonferroni's multiple comparisons test.

After having shown that adenosine through Adora2b is important to prevent bleomycin-induced acute injury, we wanted to understand whether Adora2b is also the major adenosine receptor mediating the therapeutic effect of adenosine on established acute lung injury. To study the role of Adora2b in treating acute lung injury, wildtype (*Adora2b* ^*+/+*^) or *Adora2b* ^*−/−*^ mice were subjected to bleomycin injury, followed by i.p injection of BAY 60-6583 starting on day 2 and continuing daily for 5 days (Figure 5A). BAY 60-6583 dramatically attenuated bleomycin-induced lung injury by reducing vascular leakage (Figure 5A) and inflammation (Figure 5B,C) in wildtype mice. However, the protection of BAY 60-6583 was completely abolished in the absence of Adora2b (Figure 5A–C). Moreover, *Adora2b* ^*−/−*^ mice exhibited increased vascular leakage and inflammation compared to *Adora2b* ^*+/+*^ mice (Figure 5A–C). Differential assay indicated a significant increase in neutrophil trafficking into the lungs in *Adora2b* ^*−/−*^ mice compared to wildtype controls, and the inhibition of BAY 60-6583 on neutrophil infiltration that we observed in wildtype mice was completely eliminated in *Adora2b* ^*−/−*^ mice (Figure 5D). To visualize neutrophil trafficking into the lungs, we stained the lung sections with anti-Neutrophil antibody NIMP-R14. We found the number of neutrophils was significantly increased when Adora2b was deficient (Figure 5E,F), and BAY 60-6583 dramatically inhibited neutrophil infiltration in *Adora2b* ^*+/+*^ mice but not in *Adora2b* ^*−/−*^ mice (Figure 5E,F).

**FIGURE 6:**
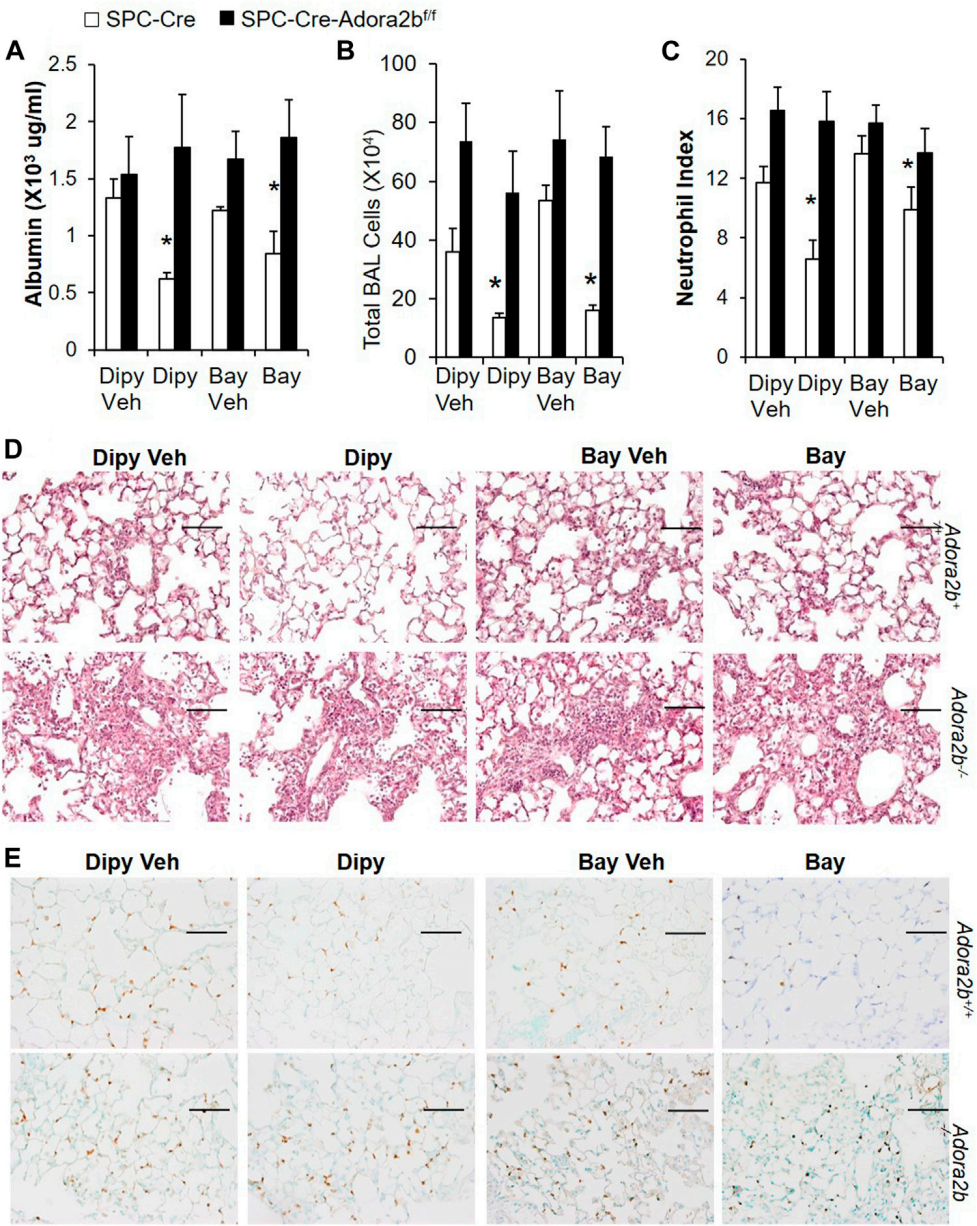
Effect of Dipyridamole and BAY 60-6583 on established ALI in mice with Adora2b knockout specifically in alveolar epithelial cells (SPC-Cre-Adora2blox/lox). *SPC-Cre* and *SPC-Cre-Adora2blox/lox* mice were treated with 2.5 U/kg bleomycin through i.t. Two days later, mice were treated with 5 mg/kg dipyridmole twice daily or 500 ug/kg BAY 60-6583 daily until day 7. BAL albumin **(A)** and total BAL cells **(B)** were measured. **(C)** Lung sections were stained with anti-Ly6B.2 for neutrophil index. **(D)** Representative H&E staining showed pulmonary inflammation and injury in various groups. **(E)** Representative lung sections stained with Ly6B.2 to show neutrophil infiltration. n = 5, *p < 0.05 vs. vehicle using one way ANOVA followed by Bonferroni's multiple comparisons test. Scale bar = 100 µm.

We have shown that epithelial Adora2b is important to prevent bleomycin-induce lung injury. Next, we wanted to determine whether epithelial Adora2b is also important for the treatment of established ALI. SPC-Cre or SPC-cre-*Adora2b* ^*f*/f^ mice were i.t. administrated with bleomycin. After 2 days, mice were i.p injected with BAY 60-6583 or dipyridamole for five consecutive days. SPC-Cre mice treated with either BAY 60-6583 or dipyridamole had better lung recovery with ameliorated vascular leakage (Figure 6A), inflammation (Figure 6B,D) and neutrophil infiltration (Figure 6C,E). However, neither BAY 60-6583 nor dipyridamole attenuated bleomycin-induced lung injury in mice lacking Adora2b in alveolar epithelial cells (Figure 6A–C). Taken together, our findings suggest that epithelial Adora2b is important for lungs to recover from bleomycin-induced acute injury.

### Adenosine Through Adora2b Maintains Epithelial Integrity by Preventing the Degradation of Junction Proteins

**FIGURE 7:**
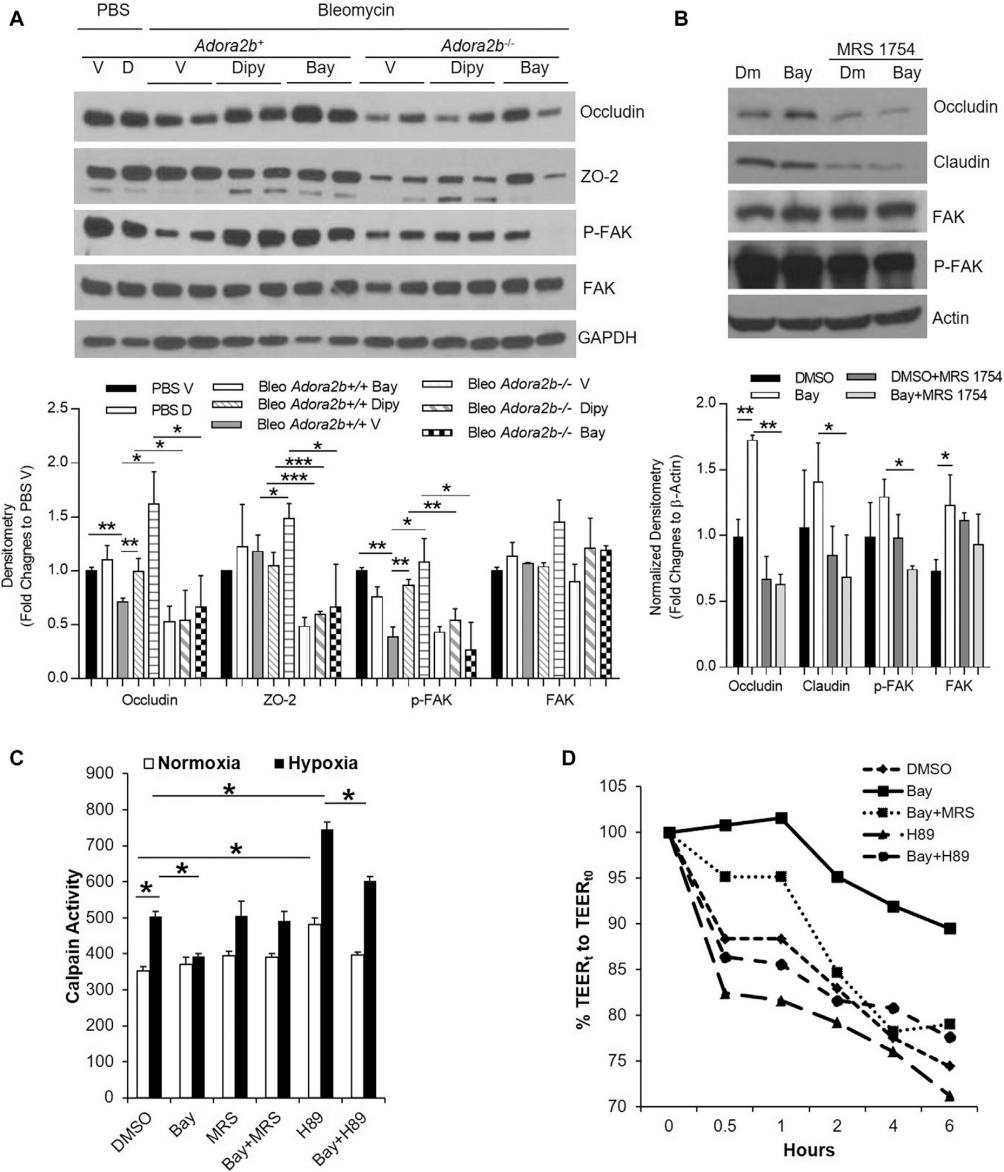
The regulation of Adora2b on junction proteins. *Adora2b* ^*+/+*^ or *Adora2b* ^−/-^ mice were **I**.t injected with 2.5 U/kg bleomycin. Two days later, mice were treated with 5 mg/kg dipyridmole twice daily or 500 ug/kg BAY 60-6583 daily until day 7 for analysis. **(A)** Upper panel: whole lung samples were collected from different treatment group for western blot analysis of Occludin and ZO-2 or phosphorylated FAK (*p*-FAK) and FAK. Lower panel: The Densitometry of the Western Blot image were analyzed. Data were presented as Fold Change to PBS Vehicle± SD. n ≥ 4, *p < 0.05, **p < 0.01. ***p < 0.001. **(B, D,E)** MLE12 cells were serum starved overnight, pretreated with ADA for 30 min, treated with 500 nM BAY 60-6583 or 1 µM MRS 1754 or 10 μM H89, and then exposed to 2% oxygen for 6 h for western blot and 1 h for Calpain activity. **(B)** Upper panel: Expression of Occludin and Claudin-1, and *p*-FAK and FAK were examined using western blot. Lower Panel: Densitometry of the Western Blot image were analyzed. Data were presented as Normalized fold changes to β-Actin ± SD. *p < 0.05, **p < 0.01. **(C)** Calpain activities were measured and quantitated. *p < 0.05 one way ANOVA followed by Bonferroni’s multiple comparisons test vs. DMSO control. **(D)** RLE-6TN cells were serum starved overnight, pretreated with ADA for 30 min, treated with 500 nM BAY 60-6583 or 1 µM MRS 1754 or 10 μM H89, and then exposed to 2% oxygen for 0–6 h, transepithelial electrical resistance (TEER) were measured and normalized to 0 h n = 3 independent experiments.

Based on the above findings, we know that adenosine through Adora2b is important not only to prevent but also to treat acute lung injury. However, the underlying mechanisms are not known. The lung epithelial monolayer is assembled through the interactions of numerous structural proteins, including occludins, claudins and focal adhesion kinase (FAK). Evidence has suggested that deteriorated expression of these proteins in epithelial cells promotes the permeability of the alveolar epithelium and facilitates the progression of ALI. To understand whether adenosine signaling affects the structural proteins, we first characterized the expression of these structural proteins in bleomycin-induced lung injury in different treatment groups. As shown in Figure 7A, the levels of occludin and phosphorylated FAK (*p*-FAK) were decreased in wildtype mice exposed to bleomycin for 7 days. Interestingly, both dipyridamole and BAY 60-6583 recovered the levels of occludin and pFAK to about the same levels as uninjured controls. Moreover, if Adora2b was absent (*Adora2b* ^−/-^ mice), the expression of *p*-FAK was more significantly decreased by bleomycin, and dipyridamole and BAY 60-6583 could no longer recover occludin and *p*-FAK levels (Figure 7A). In addition, the tight junction protein ZO-2 is highly dysregulated in Adora2b^−/−^ mice, representing massive loss of epithelial integrity in *Adora2b* ^−/−^ mice. However, dipyridamole failed and BAY 60-6583 only slightly recover the ZO-2 levels in wildtype mice, indicating that ZO-2 might not directly regulated by the Adenosine through Adora2b receptors (Figure 7A). Taking together, these results suggest that adenosine through Adora2b may directly regulate the levels of occludin-1 and *p*-FAK.

To determine whether adenosine through Adora2b directly regulates the levels of occludin and *p*-FAK in epithelial cells, we pretreated MLE12 cells, an immortalized alveolar epithelial type II cell line, with BAY 60-6583 and incubated in 2% oxygen for 6 h. BAY 60-6583 significantly increased the levels of occludin and the effect of BAY 60-6583 on occludin could be fully blocked by MRS 1754, an Adora2b antagonist (Figure 7B). Although levels of *p*-FAK and claudin-1 was unaffected by BAY 60-6583, they are largely suppressed in cells treated with MRS 1754 (Figure 7B), suggesting a role of adenosine through Adora2b in protecting the protein levels of FAK and claudin-1 under hypoxia exposure. Overall, these findings suggest that a role of Adora2b in regulation of occludin, FAK and *p*-FAK.

Since both occludin and FAK could be degraded by calpain, a protease which can be activated in acute lung injury and under stress conditions such as hypoxia (Liu et al., 2012), we hypothesized that adenosine through adora2b may maintain occludin and FAK levels in hypoxia and ALI by inhibiting calpain activity. To test this, we measured calpain activity in MLE12 cells pre-treated with BAY 60-6583 and then exposed them to hypoxia for 1 h. Calpain activity was significantly increased after hypoxia exposure and was dramatically inhibited by BAY 60-6583 (Figure 7C). The inhibition of BAY 60-6583 could be neutralized by MRS 1754 (Figure 7C). Calpain activity has been shown to be repressed by protein kinase A (PKA), a kinase that can be activated by adenosine through Adora2b. To determine whether BAY 60-6583 can suppress calpain activity through PKA activation, we treated MLE12 cells with H89, a PKA inhibitor. H89 significantly increased calpain activity, even in the presence of BAY 60-6583 (Figure 7C). Moreover, to examine whether calpain activity is directly correlated with epithelial leakage, we measured transepithelial resistance (TEER) in cells treated with different compounds and exposed to 2% oxygen for 6 h. Since MLE-12 cells can not form monolayer, we chose RLE-6TN for the TEER measurement. As shown in Figure 7D, the TEER is negatively correlated with the calpain activity shown in Figure 7C (BAY 60-6583 slowed down hypoxia-induced TEER loss, while H89 dramatically accelerated the decrease in TEER compared to control). Moreover, the protection of BAY 60-6583 was attenuated by both MRS 1754 and H89. Taken together, our data provide a novel mechanism for Adora2b-mediated protection in epithelial leakage and demonstrate that adenosine through Adora2b may attenuate occludin and FAK degradation in ALI by inhibiting calpain activity through PKA activation.

### Levels of Adenosine Signaling Proteins and Structural Proteins are Differently Expressed in Human ARDS Lungs

**FIGURE 8:**
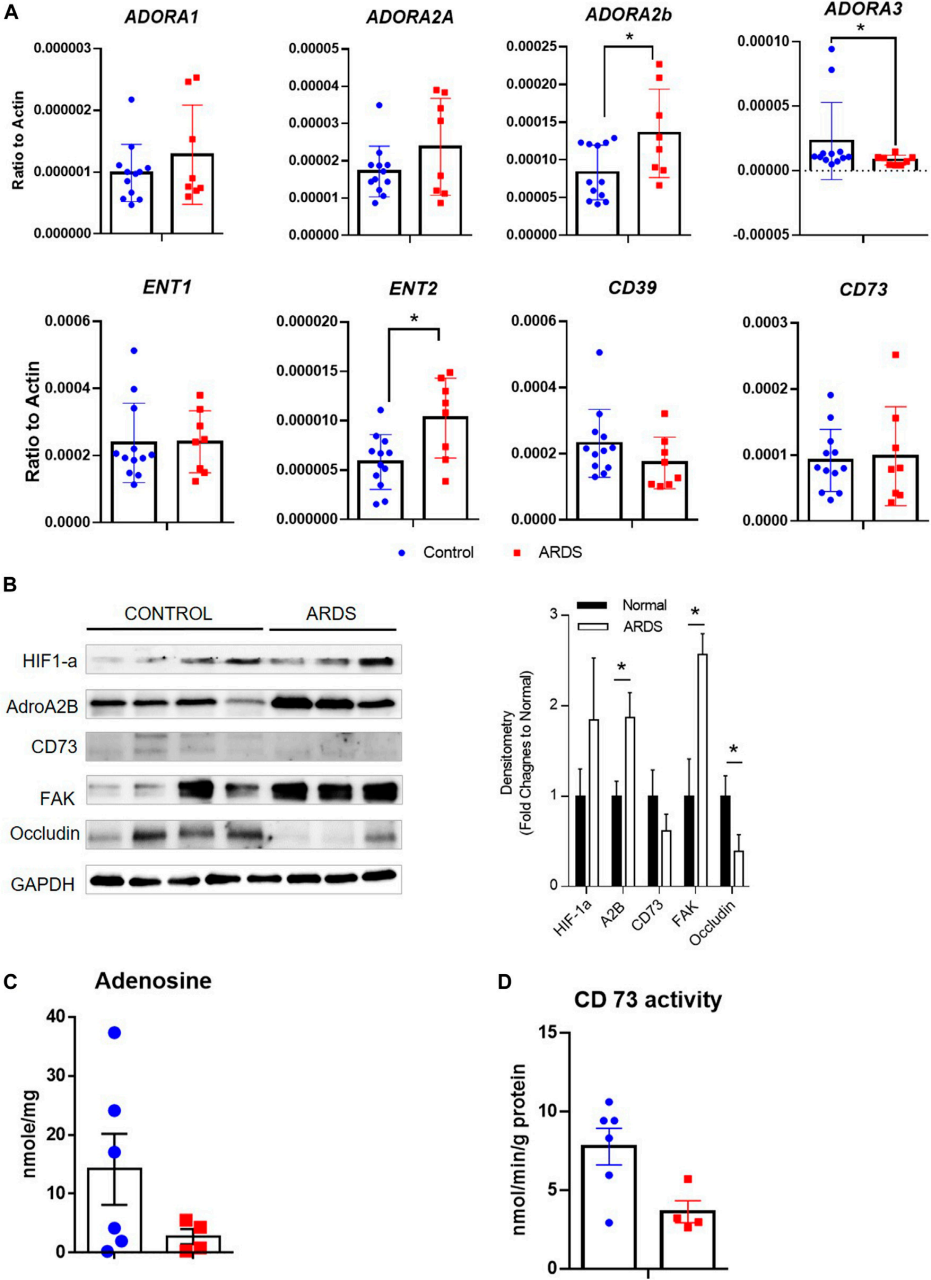
Expression of adenosine receptors, transporters and enzymes in human ARDS lungs. **(A)** The transcript expression of adenosine receptors (ADORA1, ADORA2A, ADORA2B and ADORA3), transporters (ENT1 and ENT2) as well as enzymes (CD39 and CD73) were determined in human normal and ARDS lungs using realtime PCR. **(B)** Left Panel: The protein expression of HIF-1a, ADORA2B, CD73 as well as tight junction proteins were determined in normal and ARDS lungs using western blot. Right Panel: Densitometry of the Western Blot image were analyzed and data were presented as fold changes to Normal control ± MSE. *p < 0.05. **(C)** The levels of adenosine were measured using HPLC. **(D)** The activities of CD73 were determined in control and ARDS lungs. *p < 0.05 using student t-test.

Lastly, to understand whether similar adenosine signaling is activated in human ARDS lungs, we examined the levels of adenosine receptors and transporters, as well as enzymes for adenosine synthesis in human normal and ARDS lungs. Consistent with the important roles of Adora2b in acute lung injury in mice, *ADORA2B was* the only adenosine receptor having elevated transcript and protein levels in ARDS lungs, interestingly ADORA3 levels were attenuated in ARDS lungs (Figure 8A,B). Surprisingly, *ENT2* levels were also increased in ARDS lungs (Figure 8A) in association with reduced adenosine levels (Figure 8C) and decreased CD73 activities (Figure 8D). Moreover, the levels of tight junction protein occludin was decreased in ARDS lungs compared to normal lungs (Figure 8B), suggesting that lost epithelial barrier function is also present in human ARDS lungs. Taken together, our data suggest failed adenosine accumulation in response to injury in ARDS lungs may be the potential reason for dampened barrier functions and severe pulmonary damage in ALI. .

## Discussion

**FIGURE 9:**
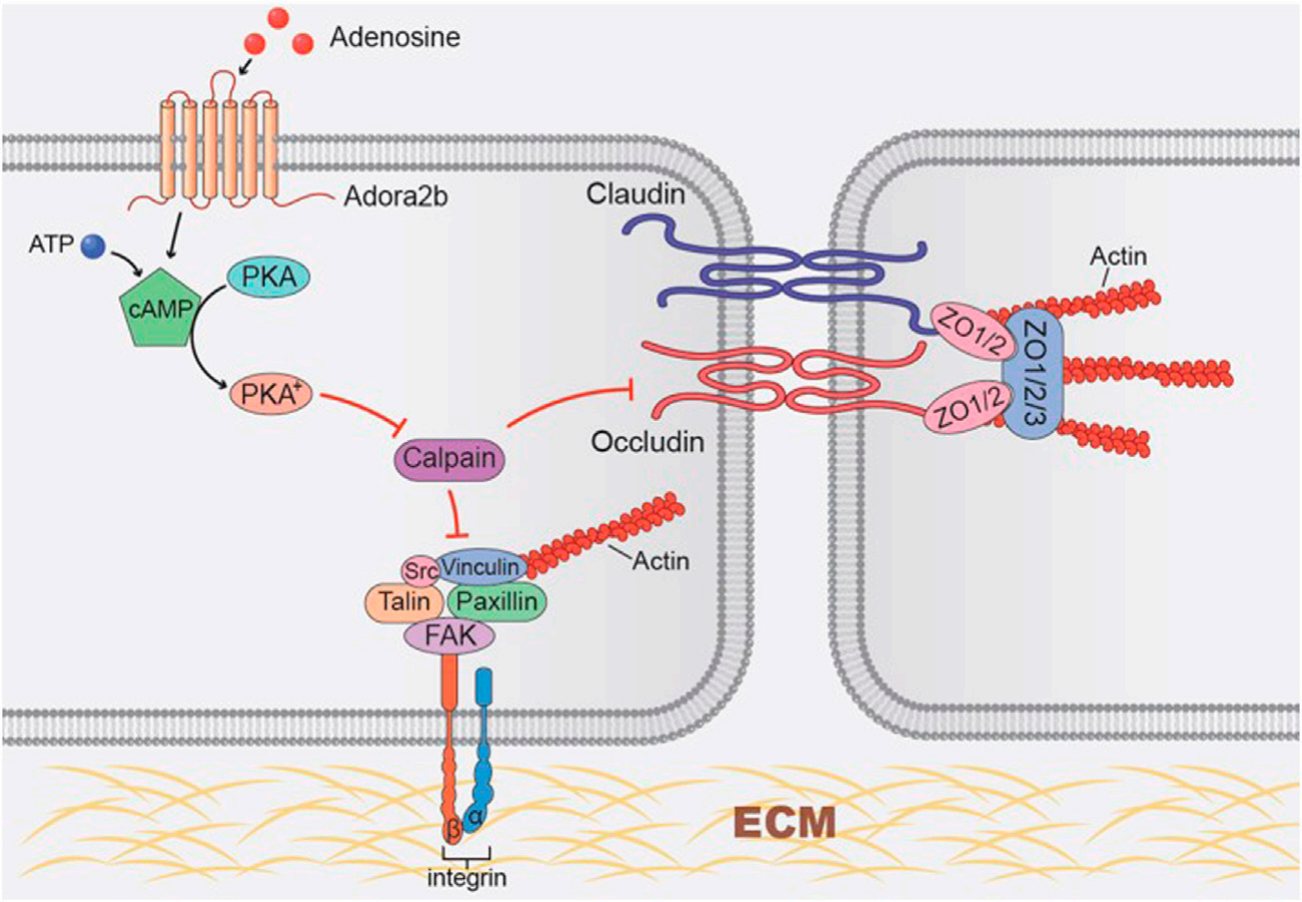
Working model of Adora2b activation in regulating tight junction and focal adhesion junction proteins. Adora2b activation increases cAMP levels by activation of adenylyl cyclase, thus activated protein kinase A (PKA). Activated PKA then inhibits hypoxia-induced calpain activity, protects occludin and FAK degradation, and maintains the epithelial integrity.

ARDS is an inflammatory lung disease that is characterized with noncardiogenic pulmonary edema, massive lung inflammation and severe hypoxemia (Bakowitz et al., 2012). Due to the limited clinical management of this disorder, ALI is among the leading causes of death in intensive care units (Bakowitz et al., 2012). This has been overemphasized in the recent COVID-19 pandemic where patients present with rapid-onset ARDS accompanied by a cytokine release storm, severe hypoxemia (Goh et al., 2020) accounting for mortality rates of up to 88.1% (Richardson et al., 2020) Therefore, novel therapeutic approaches targeted to dampen lung inflammation and pulmonary edema during ALI are urgently needed. The release of adenosine from injury tissue has been highlighted as an endogenous mechanism for tissue protection in several murine models of ALI, including ALI induced by high-pressure mechanical ventilation, lipopolysaccharide (LPS) and bleomycin (Eckle et al., 2009; Karmouty-Quintana et al., 2013). Our study implicates the role of adenosine transport inhibitor dipyridamole, which prevents adenosine re-uptake and enhances extracellular adenosine levels, in tissue protection from bleomycin-induced ALI. We observed that dipyridamole play a therapeutic role in bleomycin-induced ALI. Subsequent studies using epithelial specific Ent2 knockout mice, we demonstrated that epithelial Ent2 is important to regulate extracellular adenosine levels. Moreover, we identified epithelial Adora2b as the major adenosine receptor contributing to the protective effects of dipyridamole, and occludin and focal adhesion kinase (FAK) as potential targets of adenosine through Adora2b, thus providing a novel mechanism for adenosine-mediated barrier protection (Figure 9). Taken together, we have highlighted a role of adenosine signaling in preventing or treating ALI and identified Ent2 and Adora2b as key mediators in establishing pulmonary protection from bleomycin-induced ALI.

Previous studies identified adenosine as an endogenous protective molecule during lung injury (Eckle et al., 2009; Karmouty-Quintana et al., 2013). In response to tissue injury, ATP and ADP are released from injured cells and quickly metabolized to adenosine monophosphate (AMP) in the extracellular space. Amp is then converted to adenosine by ecto-5′-nucleotidase (CD73). Equilibrative nucleoside transporters can re-uptake extracellular adenosine which allows adenosine to freely cross the extracellular cell membrane according to its concentration gradient (Grenz et al., 2011; Eltzschig et al., 2012; Eckle et al., 2013). Dampened ALI was observed using strategies aiming to inhibit extracellular adenosine levels, including genetic deletion of CD73 or CD39 (Grenz et al., 2007; Hart et al., 2011; Hasko et al., 2011) or treating mice with adenosine deaminase (ADA) which rapidly converts adenosine to inosine. On the other hand, enhancing extracellular adenosine signaling by preventing adenosine re-uptake through ENTs attenuates ALI (Eckle et al., 2013; Morote-Garcia et al., 2013). Our findings are consistent with these studies by targeting ENT as a therapeutic method for the treatment of bleomycin-induced ALI. Inhibiting ENTs with the pharmacological agent dipyridamole ameliorates established lung injury in association with enhanced extracellular adenosine concentrations. Similar protection was also observed in mice with selective deletion of *Ent2* specifically in alveolar epithelial type II cells. Dipyridamole is a FDA-approved medication used as a vasodilator for the treatment of blood clot formation and stroke (De Schryver et al., 2007; Sacco et al., 2008). Compared to mice ENTs, dipyridamole has a more potent inhibition effect on human ENTs (Yao et al., 1997), suggesting that dipyridamole could be an even more beneficial drug in the treatment of patients by lowering the off-target effects.

In this study, we identified Adora2b as the major adenosine receptor involved in the protective effect of dipyridamole mediated lung protection. Adenosine signals through four G-protein coupled receptors (Adora1, Adora2a, Adora2b and Adora3) (Hasko et al., 2008; Blackburn et al., 2009; Eltzschig, 2009; Karmouty-Quintana et al., 2013). Adora2b plays a beneficial role in tissue protection during acute injuries (Csoka et al., 2010; Eltzschig et al., 2009b; Grenz et al., 2012b). The expression levels of Adora2b is elevated as an adaption to hypoxia and inflammation, and it in turn activates a cascade of genes which are implicated in promoting barrier function, attenuating vascular leakage, and preventing inflammation and neutrophil infiltration (Eckle et al., 2009; Koeppen et al., 2011). A study by Eckle and colleagues also suggested that Adora2b promotes fluid clearance from the alveolus and attenuates pulmonary edema (Eckle et al., 2008). Consistent with previous findings, we observed attenuated ALI in mice administrated with Adora2b agonist BAY 60-6583 and enhanced ALI in mice with global deletion of Adora2b. Since Adora2b expression is mainly observed in alveolar epithelial type II cells (Cagnina et al., 2009; Eckle et al., 2013), we also generated mice with Adora2b specifically knocked out in alveolar epithelial cells and detected a similar level of lung injury comparable to global Adora2b knockout mice and loss of protection from dipyridamole and BAY 60-6583. Our studies provide important proof for BAY 60-6583 as a therapeutic medicine for the treatment of ALI.

Previous studies provide proof of concept for adenosine signaling in adaption to hypoxia and inflammation in a wide range of injury models (Eltzschig and Carmeliet, 2011; Eltzschig et al., 2014). In response to hypoxia, transcription factor hypoxia-inducible factor (HIF) is stabilized, which subsequently activates several components of the adenosine signaling pathway, including CD39 and CD73 which elevate extracellular adenosine generation (Eltzschig et al., 2009a; Synnestvedt et al., 2002; Thompson et al., 2004) and Adora2b which enhances the protective adenosine signal (Kong et al., 2006; Eltzschig and Carmeliet, 2011; Eckle et al., 2014; Eltzschig et al., 2014). Moreover, hypoxia also suppresses ENT1 and ENT2 expression by promoting the binding of HIF to their promoters, thus inhibiting adenosine re-uptake and enhancing adenosine downstream signaling events (Eltzschig et al., 2005; Eckle et al., 2013; Morote-Garcia et al., 2013). Consistent with these studies, we observed HIF-1a stabilization following bleomycin exposure in association with a decreased ENT2 expression in the lung. Dampened ENT2 expression is also observed in primary alveolar epithelial cells exposed to hypoxia or HIF-1a stabilizer CoCl_2_, suggesting the observed down-regulation of ENT2 in ALI could be directly regulated by hypoxia. Taken together, our data provide evidence that the hypoxia-adenosine response is an important mechanism mediating the endogenous protection of ALI. Surprisingly, contrary to the enhanced adenosine levels in response to ALI in mice, we detected decreased adenosine levels in the lungs of ARDS patients. The decreased adenosine levels could be resulted from decreased CD73 activities and elevated adenosine transportation and elimination by ENT2 in ARDS lungs. It is important to mention that the ARDS lungs were collected from patients that succumbed to ARDS, thus it is conceivable that in these patients, insufficient accumulation of adenosine may have been a factor leading to their demise.

Although the role of extracellular adenosine signaling in inflammation has been explored to some extent (Eltzschig, 2011; Eltzschig and Carmeliet, 2011; Eltzschig et al., 2013; Idzko et al., 2014), the downstream mechanisms that mediate the role of adenosine in preventing epithelial permeability is not well studied. The integrity of the epithelial monolayer is critical for the protection of barrier-function (Kirchner et al., 2005; Lucas et al., 2009). Compared to endothelial damage, epithelial damage has more adverse effects on lung injury due to the destruction of the lung architecture that leads to prolonged changes in gas exchange and disordered repair (Martin T. R. et al., 2005). Studies show that preserved epithelial barrier function is inversely associated with mortality of patients with ALI (Martin T. R. et al., 2005; Matthay and Wiener-Kronish, 1990). These conclusions are consistent with our findings that Adora2b in epithelial cells, but not endothelial cells, prevents vascular leakage. Several tight junction and focal adhesion proteins are involved to maintain the integrity of epithelium, including occludins, claudins and focal adhesion kinase (FAK) (Ma et al., 2013; Suzuki, 2013). Deregulation of these proteins in epithelial cells promotes the permeability of the epithelium and facilitates the progression of injury (Jiang et al., 1998; Koval, 2009; Ma et al., 2013). Under stress conditions, cellular permeability can be accelerated by the activation of calpain protease which promotes the degradation of FAK and tight junction proteins and disrupts the cell-cell connection (Chun and Prince, 2009; Heijink et al., 2012; Pereira et al., 2014; Wang et al., 2012). In our study, we found the levels of tight junction protein occludin was decreased in both human ARDS lungs and mouse lungs treated with bleomycin, suggesting a loss of epithelial barrier function in both human and mouse ALI. However, the expression of FAK is elevated in ARDS lungs possibly due to increased infiltration of inflammatory cells such as T lymphocyte and macrophages that also have high FAK expression (Chapman and Houtman, 2014; Hart et al., 2011). We also discovered that adenosine-through Adora2b inhibits hypoxia-induced calpain activation through the activation of cyclic AMP (cAMP) and protein kinase A (PKA), and as a result prevents occludin-1 degradation and epithelial permeability (Figure 9). Indeed, under hypoxia condition, our data showed that BAY 60-6583 significantly increased the levels of occludin in MLE12 cells, and the effect of BAY 60-6583 on occludin could be fully blocked by Adora2b antagonist MRS 1754. Although levels of *p*-FAK and claudin-1 was unaffected by BAY 60-6583, they were largely suppressed in cells treated with MRS 1754 (Figure 7B), suggesting a role of adenosine through Adora2b in protecting the hypoxia-induced protein degradation of FAK and Claudin-1. FAK protein may be relatively more stable under hypoxia condition, that is probably why it can be slightly upregulated in Bay 606583 treated cells but not obviously affected by MRS 1754. Our results are consistent with previous findings showing improved ALI in mouse treated with a calpain inhibitor (Cuzzocrea et al., 2000; Liu et al., 2012). Although further studies are needed to understand how calpain is activated by hypoxia, and which calpain is directed targeted by adora2b/PKA, our findings identified calpain as a novel downstream target of Adora2b and provide evidence for the development of drugs aimed at targeting adenosine signaling and calpain as a therapeutic treatment of ALI.

In addition to providing evidence for the role of adenosine signaling in preventing ALI, our studies also identified dipyridamole and BAY 60-6583 as therapeutic agents in the treatment of established ALI. Previous studies showed a beneficial role of adenosine in a wide range of acute injury models, including sepsis (Csoka et al., 2010), and lung (Zhou et al., 2011; Eckle et al., 2013), kidney (Bauerle et al., 2011; Grenz et al., 2012b) and gastrointestinal injuries (Eltzschig et al., 2009b). However, all of these studies initiate adenosine treatment before inducing tissue injury and implicate only the prophylactic role of adenosine signaling in ALI. Here we demonstrate that treating mice with dipyridamole or BAY 60-6583 two days after bleomycin exposure, a time point when ALI is already established, still improve the barrier function by decreasing inflammation and neutrophil infiltration. Our findings are of particular importance for the treatment of ARDS patients who have already developed symptoms of ALI in the intensive care unit, and provide important preclinical proof for enhancing adenosine signaling as a therapeutic method for the treatment of ARDS patients.

Taken together, our findings identified adenosine signaling as an important endogenous pathway to not only prevent but also treat ALI. Inhibiting adenosine re-uptake by pharmaceutical agent dipyridamole could be a therapeutic strategy to enhance adenosine signaling through Adora2b that in turn prevents calpain activity and preserves epithelial integrity. Our results provide important pre-clinical data for the use of dipyridamole and adenosine receptor agonists in the treatment of ALI.

## Data Availability

The raw data supporting the conclusions of this article will be made available by the authors, without undue reservation.

## Abbreviations

This article was submitted to Molecular Diagnostics and Therapeutics, a section of the journal Frontiers in Molecular Biosciences
